# Establishment of an efficient regeneration and *Agrobacterium transformation* system in mature embryos of calla lily (*Zantedeschia* spp.)

**DOI:** 10.3389/fgene.2022.1085694

**Published:** 2022-12-06

**Authors:** Xuan Sun, Yi Wang, Tuo Yang, Xue Wang, Huanxiao Wang, Di Wang, Hongyan Liu, Xian Wang, Guojun Zhang, Zunzheng Wei

**Affiliations:** ^1^ Hebei Key Laboratory of Horticultural Germplasm Excavation and Innovative Utilization, College of Horticultural Science and Technology, Hebei Normal University of Science and Technology, Qinhuangdao, China; ^2^ Institute of Grassland, Flowers and Ecology, Beijing Academy of Agriculture and Forestry Sciences, Beijing, China; ^3^ College of Horticulture, China Agricultural University, Beijing, China; ^4^ Hebei Higher Institute Application Technology Research and Development Center of Horticultural Plant Biological Breeding, Qinhuangdao, China

**Keywords:** calla lily, regeneration system, genetic transformation, CNTs, seed embryo

## Abstract

Calla lily (*Zantedeschia* spp.) have great aesthetic value due to their spathe-like appearance and richness of coloration. However, embryonic callus regeneration is absent from its current regeneration mechanism. As a result, constructing an adequate and stable genetic transformation system is hampered, severely hindering breeding efforts. In this research, the callus induction effectiveness of calla lily seed embryos of various maturities was evaluated. The findings indicated that mature seed embryos were more suitable for *in vitro* regeneration. Using orthogonal design experiments, the primary elements influencing *in vitro* regeneration, such as plant growth regulators, genotypes, and nanoscale materials, which was emergent uses for *in vitro* regeneration, were investigated. The findings indicated that MS supplemented with 6-BA 2 mg/L and NAA 0.1 mg/L was the optimal medium for callus induction (CIM); the germination medium (GM) was MS supplemented with 6-BA 2 mg/L NAA 0.2 mg/L and 1 mg/L CNTs, and the rooting medium (RM) was MS supplemented with 6-BA 2 mg/L NAA 0.7 mg/L and 2 mg/L CNTs. This allowed us to verify, in principle, that the *Agrobacterium tumefaciens*-mediated genetic transformation system operates under optimal circumstances using the *GUS* reporter gene. Here, we developed a seed embryo-based genetic transformation regeneration system, which set the stage for future attempts to create new calla lily varieties.

## Introduction

Calla lily (*Zantedeschia* spp.) is an African perennial herbaceous bulbous flower belonging to the *Zantedeschia* genus in the Araceae family. They are highly prized as ornamental plants due to their unique forms and hues. During the growth process, calla lilies are vulnerable to several biotic and abiotic challenges. Previous research has shown that salinity in the soil induced salt stress and affected calla lily development, including a decrease in plant height, leaf number, root length, shoots, and root dry matter (Figueiredo et al., 2017). Additionally, bacterial soft rot deteriorated the bulb quality of calla lily and caused significant economic losses (Li et al., 2022). Developing attractive calla lily varieties resistant to biotic and abiotic stress is an important research topic. Conventional breeding strategies, such as hybridization and mutant breeding, result in the complicated inheritance of genetic factors and require much time (Azadi et al., 2016). In contrast, genetically modified breeding (GMB) simplifies and expedites breeding by introducing one or more genes to an organism’s genome using contemporary molecular techniques (Singh et al., 2015). In addition, the implementation of GMB relies on a stable and effective genetic transformation system. In *Euphorbia pulcherrima*, regeneration based on Agrobacterium-mediated gene editing yielded transgenic plants with orange bract color (Nitarska et al., 2021). To postpone floral senescence in *Ipomoea nil* cv. “Violet” EOH1 was also modified using gene editing technology based on the genetic transformation system (Shibuya et al., 2018). Overexpression of the Fererdoxin-like protein (*PFLP*) in *Z. elliottiana* cv. “Florex Gold” yielded a soft rot-resistant variant (Yip et al., 2007). Previous research demonstrated that the transformation effectiveness of calla lily is inadequate, even though the transgenic approach is superior for assessing the molecular mechanism of a calla lily. Therefore, developing a genetic transformation and regeneration system for the calla lily remains challenging.

Somatic embryogenesis (SE), a unique phase of embryonic development, is essential to establishing a regenerative system. It is possible to generate somatic embryos directly from leaves, petals, and stem segments (Su et al., 2009) or indirectly through embryogenic callus (Von-Arnold et al., 2002). A few investigations into SE in calla lily are available. An embryogenic callus was formed from leaves, but no regenerated plants were established (Gong et al., 2008). Since then, embryogenic callus has been induced from bulbs and regenerated into whole plants, with a 25% induction efficiency (Duquenne et al., 2006; Han and Kim, 2019). Transgenic plants based on embryogenic callus induction produce fewer chimeras than those based on direct somatic embryogenesis, which is a more favorable platform for genetic transformation (Rout et al., 1991). To learn more about the genetic transformation system, developing a system by inducing embryogenic callus is necessary.

Several variables, including explant type, genotype, and plant growth regulators (PGR), might affect the process of establishing a regeneration system induced by embryogenic callus (Liu et al., 2022). The efficiency of genetic transformation is reported to be influenced by explant type (Aggarwal et al., 2011). For example, the effect is different in the leaves and internodes of *Solanum tuberosum* (Kaur et al., 2020). The initial selection of suitable explants is essential for effectively inducing embryonic callus. In *Paeonia arborea*, buds, leaves, petioles, stem tips, petals, roots, and mature embryos have been exploited for regeneration (Cerna et al., 2001; Beruto et al., 2004; Teixeira da Silva et al., 2012; Gao et al., 2020). However, with *P. ostii* cv. “Fengdan”, mature embryos were more desirable explants (Liu et al., 2022). In *Nelumbo nucifera*, the induction rate was greater when seed embryos were employed as explants than when shoot tips, cotyledons, or shoots were used (Deng et al., 2020). Furthermore, embryos have been effectively used for plant regeneration in tissue culture of wheat, rice, maize, and other extremely recalcitrant plants (Yan et al., 2010; Rostami et al., 2013; Tamimi and Othman 2021). Numerous explants have been used to regenerate calla lily plantlets so far. However, the embryogenic callus induction coefficient was still low (Duquenne et al., 2006; Han and Kim, 2019). Therefore, it is essential to develop novel explants to enhance the efficacy of embryogenic callus formation. Then, the induction impact of various genotypes in plants varied. It has been reported that *Z. aethiopica* and *Z. hybrida* (interspecies hybrids mainly generated from *Z. elliotiana*, *Z. pentlandii*, *Z. albinoscapulata*, and *Z. rehmannii*, *etc.*) have been utilized for induction, and variation in induction rates of *Z. aethiopica* was 16.3% while *Z.* hybrida was 24.4% (Jonytienė et al., 2017). In conclusion, the combination and concentration of PGRs have been thoroughly examined to evaluate their effects on several plant tissue culture regeneration processes, such as callus formation and root induction. In *P. suffruticosa*, indole-3-butyric acid (IBA) was widely utilized as a rooting-inducing PGR, typically in combination with indole-3-acetic (IAA) or naphthaleneacetic acid (NAA) (Wang et al., 2012). In calla lily, 6-benzylaminopurine (6-BA) and Thidiazuron (TDZ) are often employed. 6-BA and NAA also are the most frequent hormone combinations for inducing embryogenic callus in calla lily (Chang et al., 2003; Duquenne et al., 2006; Gong et al., 2008; Han and Kim, 2019). However, additional research is required to determine the suitable kinds and concentrations of hormones for distinct species.

In addition, nanomaterials have increasingly become the most critical component influencing *in vitro* regeneration. Nanomaterials have unique physicochemical qualities with a large surface area and high reactivity. It may interact with a range of biological systems (Asgari-targhi et al., 2018), such as stimulating seed germination (Lahiani et al., 2013), impacting plant growth and development, and so on (Siddiqui and Al-Whaibi, 2014). Carbon nanotubes (CNTs) may facilitate the development of tobacco cell cultures in nanomaterials by organizing marker genes for cell division, cell wall construction, and water transport (Khodakovskaya et al., 2012). The addition of CNTs to the medium of *Rubus glandularis* may facilitate the germination and rooting processes in tissue culture (Flores et al., 2014). While seeds were utilized as explants in *Catharanthus roseus*, adding multi-walled CNTs to the culture medium may positively influence the proliferation and differentiation of callus (McGehee et al., 2019). *C. roseus* seedlings flourished on MS medium enriched with CNTs, and leaf length, leaf area, and root length all increased substantially (Ghasempour et al., 2019). Meanwhile, the addition of CNTs in *Satureja khuzestanica* expedited callus development and secondary metabolite production (Ghorbanpour and Hadian, 2015). In contrast, adding CNTs reduced the root elongation process in tomato and lettuce (Canas et al., 2008). Therefore, various plants respond differently to CNTs. It is uncertain what function CNTs have in calla lily tissue culture.

In this work, we provided an improved and comprehensive procedure for induction and regeneration employing hybrid seed embryos as calla lily’s explants. For callus induction, germination, and rooting, various hybrid seed embryos were deposited in a medium enriched with varied concentrations of 6-BA, NAA, and CNTs. Using orthogonal experiments, the system of callus induction and regeneration of calla lily hybrid seed embryos was established. In order to execute preliminary validation of the aforementioned procedure, the callus was infected with the *GUS* reporter gene. It established the groundwork for the renovation of the calla lily genetic transformation system.

## Materials and methods

### Embryonic callus induction rate of seed embryos at different stages after pollination

After hybridization of *Zantedeschia* cv. “Black Magic” and cv. “Liming” calla lily embryo seeds were harvested at 40, 47, 54, and 61 days after pollination (DAP) and kept at 4°C for 3 days. Afterward, the seeds were soaked with detergent for 15 min and rinsed for 2 h. The seeds were transported to an ultra-clean workbench for the purpose of sterilization. The seeds were initially sterilized with 75% ethanol before being rinsed three times with sterilized water. Depending on the size of the seeds, the seeds were then disinfected with 1% NaClO for 9–15 min and washed at least three times with sterile water. The sterilized seeds’ surface water was removed, and the embryos were placed on the callus induction medium (MS medium supplemented with 2 mg/L 6-BA and 0.1 mg/L NAA). The culture was incubated in a culture room in the dark at 25°C. In this experiment, four treatments were repeated three times, and 30 seed embryos were inoculated in each treatment. During this period, embryos developed adventitious buds, and an embryonic callus was produced at the base of adventitious buds. After 3 weeks, the rate of embryonic callus induction was determined. Embryonic callus induction rate is the ratio of the number of seed embryos producing callus to the total number of seed embryos. Paraffin slices were utilized to observe embryonic callus under the microscope (Yu et al., 2019).

### Induction of embryogenic callus

A completely orthogonal methodology was used in this investigation. The genotype, 6-BA concentration, NAA concentration, and CNTs concentration components, each with five levels defined, were established (Supplementary Table S1). On each medium supplemented with varied concentrations of 6-BA (0.5, 1.0, 1.5, 2, and 2.5 mg/L), NAA (0.1, 0.2, 0.3, 0.4, and 0.5 mg/L), and CNTs (0.0, 0.05, 0.1, 0.2, and 0.4 mg/L), more than 30 seed embryos of the calla lily (cv. “Kelso” × cv. “Samba”; cv. “7G” × cv. “'Sunshine”; cv. “Kloon” × cv. “Picasso”; cv. “4 days” × cv. “Liming”; cv. “Black Magic” × cv. “Liming”) were placed to induce somatic embryogenesis. Three weeks were spent incubating those explants in total darkness. The most efficient CIM was determined by calculating the callus induction rates of seeds grown in the dark for 30 days. To induce somatic embryos, the callus was moved to an embryo induction medium (EIM) with 2 mg/L 6-BA and kept in darkness for 2 weeks while the medium was replenished twice.

### Germination and rooting of somatic embryos

An orthogonal experiment was designed to test the effects of varying concentrations of PGR and CNTs on somatic embryo germination and rooting. Three levels for each of the three components (6-BA concentration, NAA concentration, and CNTs concentration) were established (Supplementary Table S2, Supplementary Table S3). The number of germinations of each embryogenic callus was counted, and the germination rate was estimated for each treatment. Two weeks following transfer to the RM, the total number of roots and mean root length for each treatment were measured. All these were grown at 25°C with 16 h of light and 8 h of darkness.

### *A. tumefaciens*-mediated transformation of somatic embryos

The *GUS* reporter gene-containing pCAMBIA1304 vector was used to transform somatic embryos in calla lily. Heat shock-mediated transformation of the plasmid into the *A. tumefaciens* EHA105 strain (Zhang et al., 2019a). Bacteria were cultured overnight at 28°C with 50 mg/L kanamycin and 25 mg/L rifampicin with shaking (200 r/min), then centrifuged at 5,000 rpm for 8 min at room temperature until OD_600_ = 0.8. *A. tumefaciens* was suspended in a 200 mol/L acetosyringone-supplemented MS liquid medium. After gently shaking the somatic embryos for 15 min in the prepared bacterial solution, the bacterial solution is removed from the surface of the somatic embryos. The somatic embryos that had been transformed were incubated in a co-culture medium (CM) at 25°C in the dark for 3 days.

### Selection and identification of transgenic somatic embryos

Somatic embryos were moved to a selective medium (SM), which contained MS supplemented with 300 mg/L cephalosporin and 8 mg/L hygromycin, under 16 h light and 8 h dark conditions for 2 days after co-culturing. Then assess the efficacy of somatic embryo transformation and discard any browned tissue. Somatic embryos with a diameter of more than 1 cm, as well as a healthy growth status were finally selected for GUS staining (Li et al., 2016). GUS staining was used to quickly identify the effect of gene transformation. Wild-type callus was used as control. All the callus was rinsed thrice in sterile water and stained in a 2 mmol/L XGluc (5-bromo-4-chloro-3-indolyl-d-glucuronide and cyclohexylammonium salt) solution overnight at 37°C. After staining, the callus was washed in 70% ethanol. It was immersed in the predetermined staining solution for 12 h, followed by observing the dyed somatic embryos using a microscope. Transient GUS expression was documented by recording the number of explants with at least one blue focus.

### Real-time qPCR analysis

Total RNA was extracted from calli using the EASYSpin Plus polysaccharide polyphenol/complex plant RNA Rapid Extraction Kit (Beijing Aidlab Biotechnology Co., LTD.). The HiScript III 1st Strand cDNA Synthesis Kit (Nanjing Vazyme Biotechnology Co., LTD.) was used to reverse transcribe and synthesize cDNA. Real-time qPCR analysis was performed using Taq Pro universal SYBR qPCR Master Mix (Nanjing Vazyme Biotechnology Co., LTD.). The reference gene was *ZhActin* (Guttman et al., 2021), and the primers were listed in Supplementary Table S4.

### Data analysis

Microsoft Excel 2010 was used for data analysis, while SPSS 23.0 (IBM, Armonk, NY, United States) was utilized for Analysis of Variance (ANOVA), with a significance level of *p*-value ≤0.05.

## Results

### Seed maturity affects the efficiency of embryonic callus induction

**FIGURE 1:**
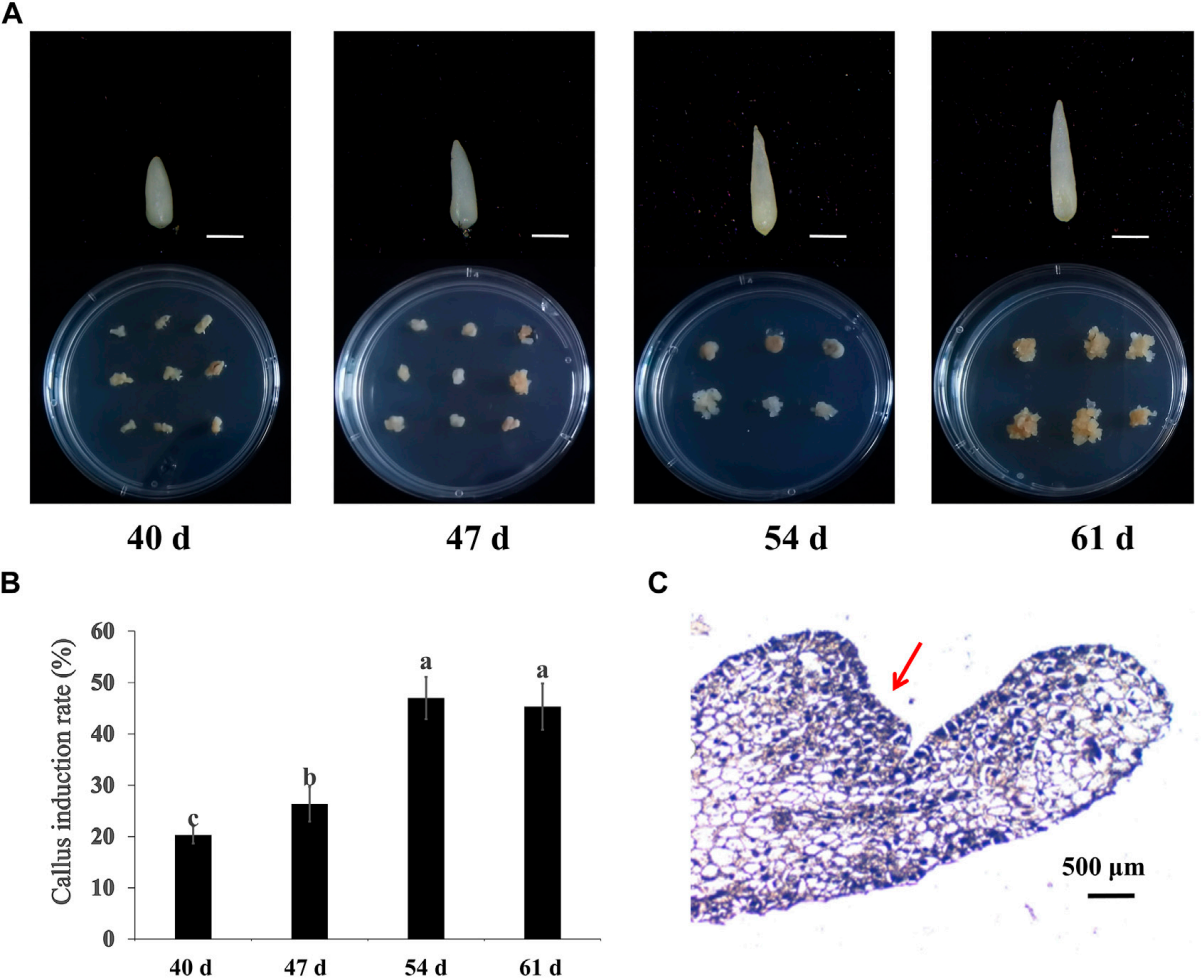
Embryonic callus induced from hybrid seeds embryo of cv. “Black Magic” × “Liming” at different maturity. **(A)** Induction of seed embryos at different stages and callus at 21 days. Scale bars, 500 μm **(B)** Callus induction rates of seed embryos of different maturity levels. **(C)** Paraffin section observation of embryogenic callus.

To generate embryonic callus, calla lily seed embryos from four specific periods (40, 47, 54, and 61 DAP) were cultivated on MS medium with 2 mg/L 6-BA and 0.1 mg/L NAA under dark conditions. Three weeks later, callus emerged at the base of seed embryos that produced adventitious buds. Figure 1A depicts the seed embryos at various developmental stages after 3 weeks of incubation. It was discovered that 61 DAP-induced embryonic calli seed embryos developed larger calli. There were statistically significant differences between the four stages (*p* < 0.05) in the rate at which seed embryos elicited callus. The average callus induction rates of seed embryos at 54 DAP and 61 DAP were not substantially different: 47% and 45%, respectively. However, low-maturity seeds were less effective in inducing callus and required more time to develop (Figure 1B **)**. This further demonstrates the significance of seed maturity for embryonic callus formation. Therefore, 61 DAP seeds were chosen as the optimal sample period for embryonic callus induction. When tissue sections were monitored, it was revealed that the callus cells were very small, with large nuclei and dense organization, consistent with embryonic callus features (Figure 1C).

### Optimization of induction conditions for embryonic callus

**FIGURE 2:**
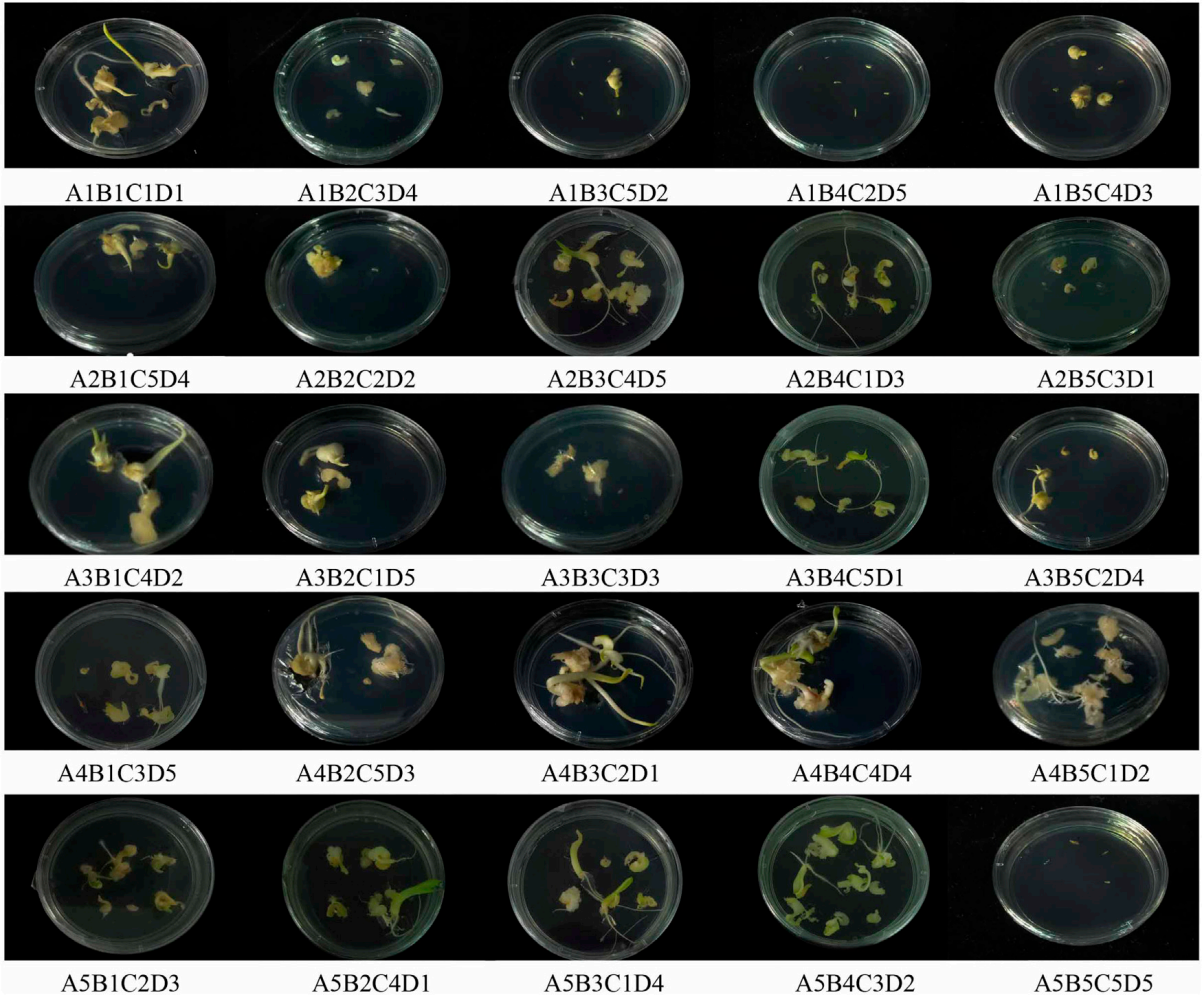
Callus induction in hybrid seeds embryo of calla lily by different orthogonal design.

An orthogonal experimental design was used to maximize the influencing variables of embryogenic callus induction. Five levels were established for each component, including genotype, 6-BA concentration, NAA concentration and CNTs concentration. Compared to others, seed embryos in the treatment A4B5C1D2 generated a greater number of advent buds and quite a larger basal callus. Additionally, no callus formation was observed under A1B4C2D5 and A5B5C5D5 (Figure 2).

**FIGURE 3:**
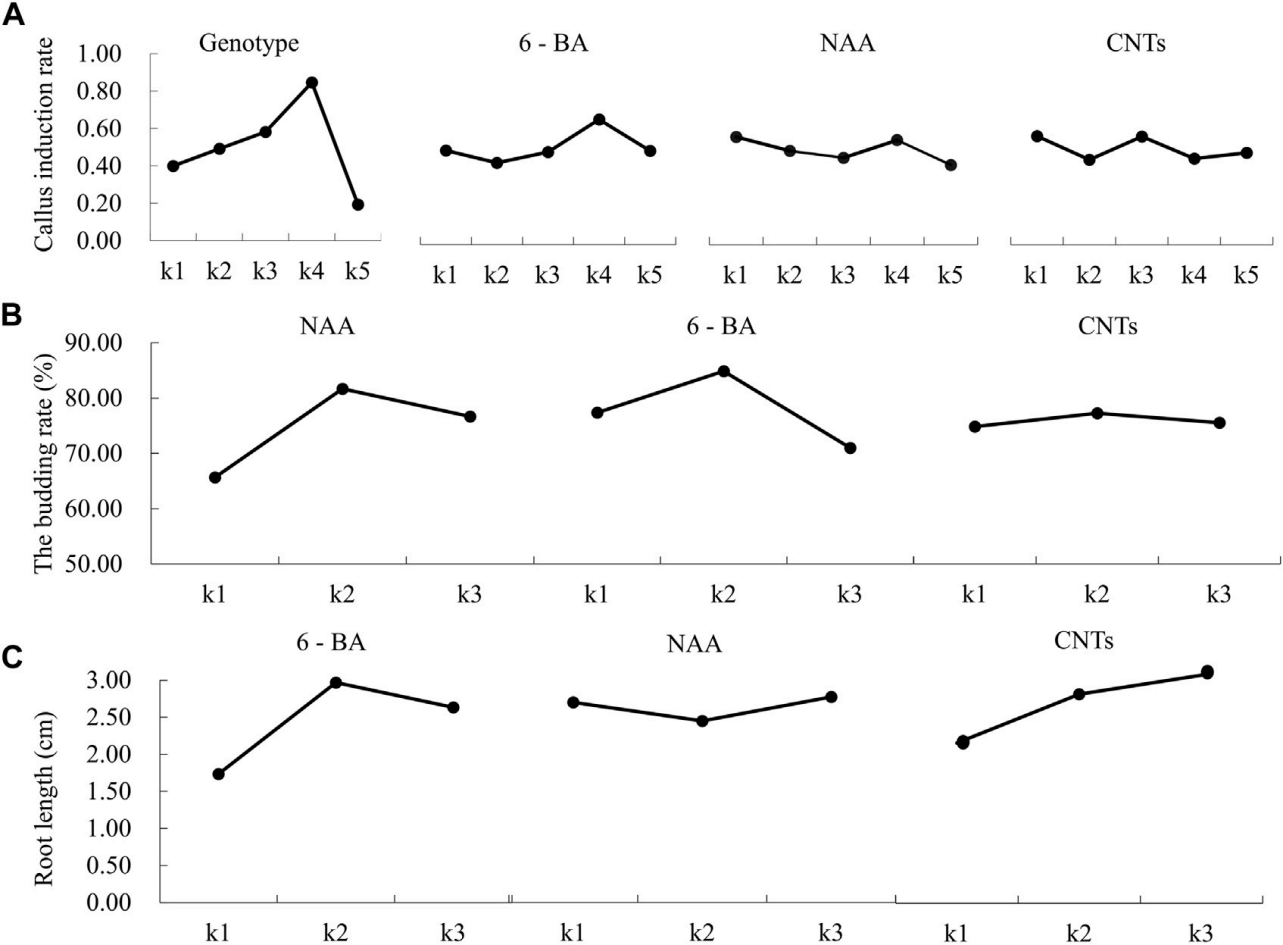
Effects of different factors on callus induction, germination, and rooting of hybrid seed embryos of calla lily. **(A)** Effects of different factors on callus induction. **(B)** Effects of different concentration of NAA, 6-BA and CNTs on callus germination. **(C)** Effects of different concentration of NAA, 6-BA and CNTs on callus rooting.

The genotype had the greatest impact on the embryogenic callus induction rate, followed by 6-BA concentration, NAA concentration and CNTs concentration (Table 1). The “Black Magic” and “Liming” hybrids exhibited the most efficient callus induction effectiveness. The amount of callus formed at the base of the adventitious bud was greater under the same culture conditions. The findings of the orthogonal experimental design indicate that the optimal cultivation condition is A4B4C1D1, namely, that the 6-BA concentration is 2 mg/L, and the NAA concentration is 0.1 mg/L. The trends between the components and the callus induction rate were analyzed based on the findings of an orthogonal experimental design (Figure 3A). As the 6-BA concentration increased, the induction rate increased and subsequently declined, reaching its maximum at 2 mg/L. In terms of NAA concentration, the induction rate declined steadily with increasing concentration, with the maximum induction rate occurring when 0.1 mg/L NAA was added. However, CNTs were deemed the least significant component, exemplifying the highest callus formation rate without addition.

**TABLE 1:** Statistical result of orthogonal experimental design for callus induction from calla lily seed embryos.

Test processing	Genotype A	6-BA concentration (mg/L) B	NAA concentration (mg/L) C	CNTs concentration (mg/L) D	Induction rate (%)
A_1_B_1_C_1_D_1_	1	0.50	0.10	0.00	66.00
A_1_B_2_C_3_D_4_	1	1.00	0.30	0.20	33.00
A_1_B_3_C_5_D_2_	1	1.50	0.50	0.05	49.00
A_1_B_4_C_2_D_5_	1	2.00	0.20	0.40	0.00
A_1_B_5_C_4_D_3_	1	2.50	0.40	0.10	52.00
A_2_B_1_C_5_D_4_	2	0.50	0.50	0.20	41.00
A_2_B_2_C_2_D_2_	2	1.00	0.20	0.05	46.00
A_2_B_3_C_4_D_5_	2	1.50	0.40	0.40	35.00
A_2_B_4_C_1_D_3_	2	2.00	0.10	0.10	69.00
A_2_B_5_C_3_D_1_	2	2.50	0.30	0.00	55.00
A_3_B_1_C_4_D_2_	3	0.50	0.40	0.05	44.00
A_3_B_2_C_1_D_5_	3	1.00	0.10	0.40	51.00
A_3_B_3_C_3_D_3_	3	1.50	0.30	0.10	54.00
A_3_B_4_C_5_D_1_	3	2.00	0.50	0.00	65.00
A_3_B_5_C_2_D_4_	3	2.50	0.20	0.20	77.00
A_4_B_1_C_3_D_5_	4	0.50	0.30	0.40	62.00
A_4_B_2_C_5_D_3_	4	1.00	0.50	0.10	63.00
A_4_B_3_C_2_D_1_	4	1.50	0.20	0.00	74.00
A_4_B_4_C_4_D_4_	4	2.00	0.40	0.20	55.00
A_4_B_5_C_1_D_2_	4	2.50	0.10	0.05	92.00
A_5_B_1_C_2_D_3_	5	0.50	0.20	0.10	14.00
A_5_B_2_C_4_D_1_	5	1.00	0.40	0.00	24.00
A_5_B_3_C_1_D_4_	5	1.50	0.10	0.20	28.00
A_5_B_4_C_3_D_2_	5	2.00	0.30	0.05	31.00
A_5_B_5_C_5_D_5_	5	2.50	0.50	0.40	0.00
K1	2.00	2.28	2.69	2.67	—
K2	2.46	1.98	2.34	2.09	—
K3	2.91	2.24	2.17	2.66	—
K4	4.23	3.03	2.61	2.12	—
K5	0.97	2.27	1.99	2.26	—
k1	0.40	0.46	0.54	0.53	—
k2	0.49	0.40	0.47	0.42	—
k3	0.58	0.45	0.43	0.53	—
k4	0.85	0.61	0.52	0.42	—
k5	0.19	0.45	0.40	0.45	—
Average	0.503	0.472	0.472	0.472	—
R	65.20	21.0	14.00	11.60	—
The sorting	A (Genotype) > B (6-BA) > C (NAA) > D (CNTs)				
Better solution	A_4_	B_4_	C_1_	D_1_	—

The most effective treatment cohort was then subjected to callus induction. The percentage of induction rate was 94%, which was greater than that of other orthologous treatments. Consequently, the orthogonal experimental design yielded observations with a high induction efficiency.

### Optimization of conditions for germination and rooting

To examine their effects on germination and rooting rate, MS medium was supplemented with various concentrations of PGRs and CNTs. Three components, each referring to three levels, were included in the orthogonal experimental design (Table 2, Table 3).

**TABLE 2:** Statistical results of orthogonal experimental design for germination rate of calla lily.

Test processing	NAA concentration (mg/L) E	6-BA concentration (mg/L) F	CNTs concentration (mg/L) G	The germination rate (%)
E_1_F_1_G_1_	0.1	1.0	0.0	57.0
E_1_F_2_G_3_	0.1	2.0	2.0	76.0
E_1_F_3_G_2_	0.1	3.0	1.0	64.0
E_2_F_1_G_3_	0.2	1.0	2.0	81.0
E_2_F_2_G_2_	0.2	2.0	1.0	82.0
E_2_F_3_G_1_	0.2	3.0	0.0	79.0
E_3_F_1_G_2_	0.3	1.0	1.0	82.0
E_3_F_2_G_1_	0.3	2.0	0.0	79.0
E_3_F_3_G_3_	0.3	3.0	2.0	63.0
K1	197.00	223.00	221.00	—
K2	245.00	243.00	228.00	—
K3	230.00	206.00	223.00	—
k1	65.67	74.33	73.67	—
k2	81.67	81.00	76.00	—
k3	76.67	68.67	74.33	—
Average	74.67	74.67	74.67	—
R	16.00	12.33	2.33	—
The sorting	F (NAA) > E (6-BA) > G (CNTs)			
Better solution	E_2_	F_2_	G_2_	—

**TABLE 3:** Statistical results of orthogonal experimental design for rooting.

Test processing	6-BA concentration (mg/L) H	NAA concentration (mg/L) I	CNTs concentration (mg/L) J	Root length (cm)
H_1_I_1_J_1_	1.0	0.3	0.0	1.5
H_1_I_2_J_3_	1.0	0.5	2.0	1.6
H_1_I_3_J_2_	1.0	0.7	1.0	2.1
H_2_I_1_J_3_	2.0	0.3	2.0	3.5
H_2_I_2_J_2_	2.0	0.5	1.0	3.1
H_2_I_3_J_1_	2.0	0.7	0.0	2.3
H_3_I_1_J_2_	3.0	0.3	1.0	2.5
H_3_I_2_J_1_	3.0	0.5	0.0	2.1
H_3_I_3_J_3_	3.0	0.7	2.0	3.3
K1	5.20	7.50	5.90	—
K2	8.90	6.80	7.70	—
K3	7.90	7.70	8.40	—
k1	1.73	2.50	1.97	—
k2	2.97	2.27	2.57	—
k3	2.63	2.57	2.80	—
Average	2.44	2.44	2.44	—
R	1.23	0.30	0.83	—
The sorting	H (6-BA) > I (CNTs) > J (NAA)			
Better solution	H_2_	I_3_	J_3_	—

NAA was the most influential factor during the germination stage, followed by 6-BA and CNTs. Following the analysis of the germination induction rate data, the optimal orthogonal experimental design combination was E2F2G2, which consisted of 2 mg/L NAA, 2 mg/L 6-BA and 1 mg/L CNTs (Table 2). The percentage of germination was 82%. In the orthogonal experimental design, each factor’s effect on the germination rate is discernible. The germination rate rose and subsequently decreased as the concentration increased for all three independent variables (Figure 3B). Based on the information above, it might be possible to figure out the optimal range of induction concentrations for each component.

During the rooting stage, the rooting effectiveness is mainly determined by the concentration of various hormones, with 6-BA, CNTs, and NAA serving the most critical roles. The optimal combination is H2I3J3, 2 mg/L 6-BA, 0.7 mg/L NAA, and 2 mg/L CNTs (Table 3). To assess the rooting impact in more detail, the rooting length of each treatment is also employed as a criterion for evaluation. The average root length under optimal growth conditions was 3.7 cm. The average root length increased initially and then decreased as 6-BA concentration rose, with the maximum average root length occurring at 2 mg/L. Intriguingly, we discovered that CNTs were advantageous for callus roots. The root length grows as CNTs concentration increases (Figure 3C). This offers a solid data foundation for nanomaterial-assisted tissue culturing.

### *A. tumefaciens*-mediated embryogenic callus transformation and identification of calla lily

**FIGURE 4:**
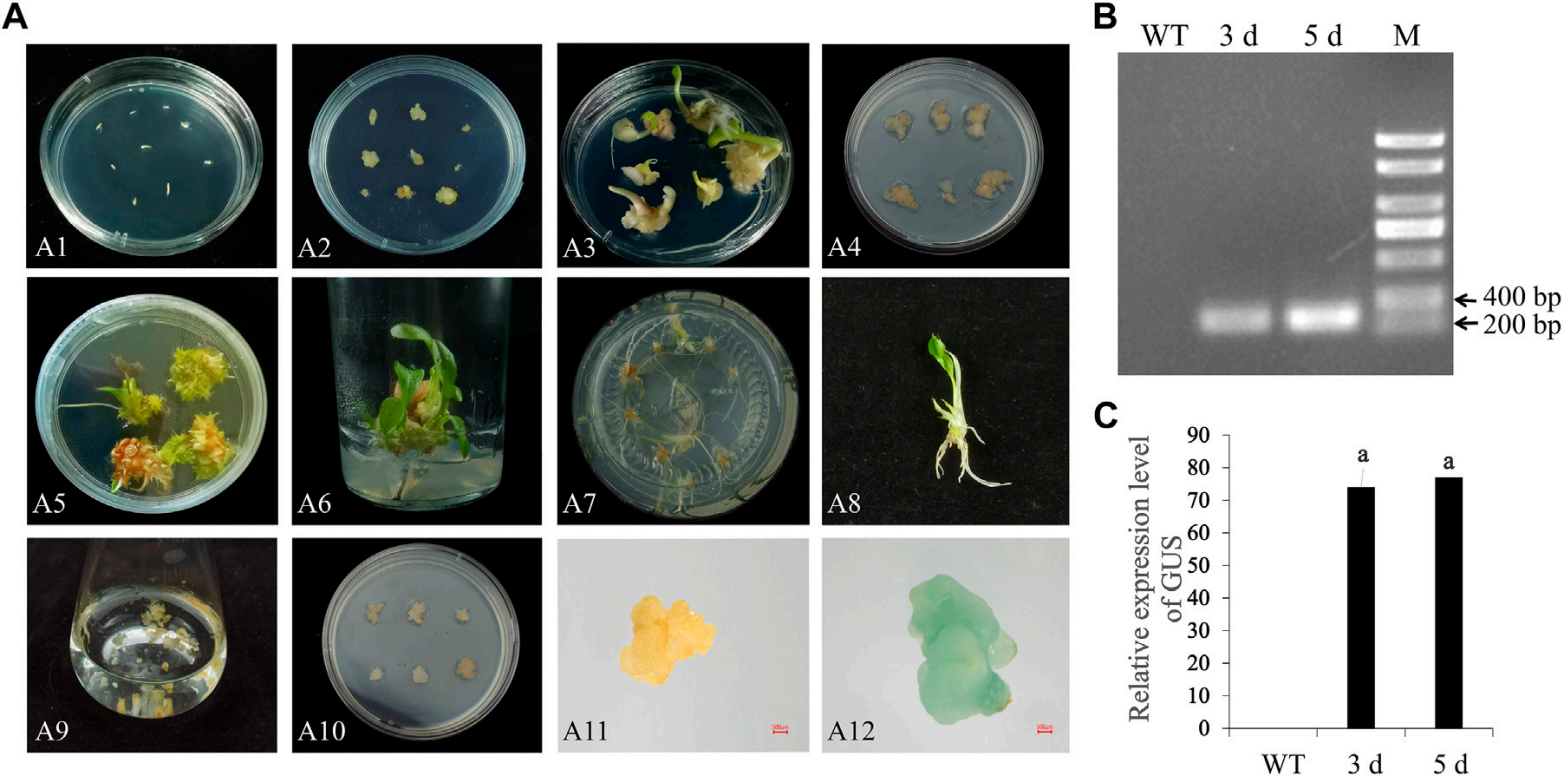
Somatic embryo induction and genetic transformation of calla lily. **(A)** A1: Seed embryo inoculation; A2: Seed embryo germination; A3: Seed embryo produces callus; A4: Callus propagation; A5: Callus germination; A6: Elongation; A7: Take root culture; A8: Intact plant; A9: Infect; A10: Co-culture; A11: *GUS* staining control group; A12: *GUS* staining test group; Scale bars, 500 μm. **(B)** Identification of transformation effect by PCR of the *GUS* fragment, and M represents the DNA marker **(C)** Identification of *GUS* expression in transformed callus.

Figure 4A depicts the process of embryogenic callus induction and plant regeneration in calla lily. The embryogenic callus derived from the seeds of a calla lily was more capable of regeneration. On CIM, the seed embryos were cultivated. After 10 days, these seed embryos grew in size with the appearance of adventitious buds. After 3 weeks of continuous culture, an abundant callus formed at the base of adventitious buds, which was changed every 2 weeks with a fresh EIM to continue proliferation. The induction of embryogenic callus occurred in circumstances of complete darkness, which aided in forming larger callus and increased the proportion of embryogenic callus. The callus underwent two rounds of culture before being transferred to GM for germination. Under light and with the optimum hormones, the callus progressively emerged green and germinated at this time. During this timeframe, weekly subcultures were conducted, and about 2 weeks later, when the leaves had completely developed, a subsequent rooting culture could be implemented. When healthy seedlings were transferred to RM, the callus progressively developed roots, and they could be transplanted into the soil around 2 weeks later. This procedure is carried out in a cycle of 16 h light and 8 h dark (Figure 4A1–8).

The embryogenic callus obtained with the above procedures is an excellent transgenic material. To further explore the genetic transformation system of calla lily, embryogenic callus transformation mediated by *A. tumefaciens* was utilized to develop a method with high transformation efficiency. *A. tumefaciens* EHA105, containing the pCAMBIA1304 (with *GUS* reporter gene) vector, was used to transform embryonic callus with a diameter of more than 1 cm and a light-yellow hue. Immerse the embryogenic callus first in an infection solution with an OD_600_ value of between 0.6 and 0.8, ensure that the solution fully covers the embryogenic callus, and gently shake it for 15 min. The callus was then put on sterile absorbent paper to remove any remaining fluids before being transferred to CM for 3 days in the dark. Excessive growth of *A. tumefaciens* will prevent callus germination at this stage. Therefore, as much extra bacterial fluid as feasible should be removed to avoid contamination. To detect transformed embryogenic callus, the surviving callus were transferred to SM containing 300 mg/L cephalosporin and 8 mg/L hygromycin for 2 days after 3 days of co-culture. However, it hindered the germination and proliferation of embryogenic callus. Thus, it needs to modify and adjust hygromycin concentration to achieve an equilibrium between selection efficiency and germination. After transformation 5 days, GUS staining was performed on the embryogenic callus to evaluate and further assess the transformation efficacy. Figure 4A9–12 show that the *GUS* reporter gene was successfully expressed in the embryogenic callus, but not in the wild type. This indicated that the genetic transformation method worked. The results showed that one sixth of the callus was stained, which proved that *A. tumefaciens* could effectively mediate the transformation of callus of *Zantedeschia*.

To further validate the impact of the vector’s transformation, the *GUS* gene was amplified using specific primers. PCR products of the *GUS* gene (329 bp) were amplified from callus gDNA (Figure 4B). RT-qPCR results revealed that *GUS* gene expression was not detected in the wild type but in infected embryonic callus. On days 3 and 5, after the transformation, the *GUS* expression level rose dramatically, which indicated that the process of transformed the genes worked (Figure 4C).

## Discussion

**FIGURE 5:**
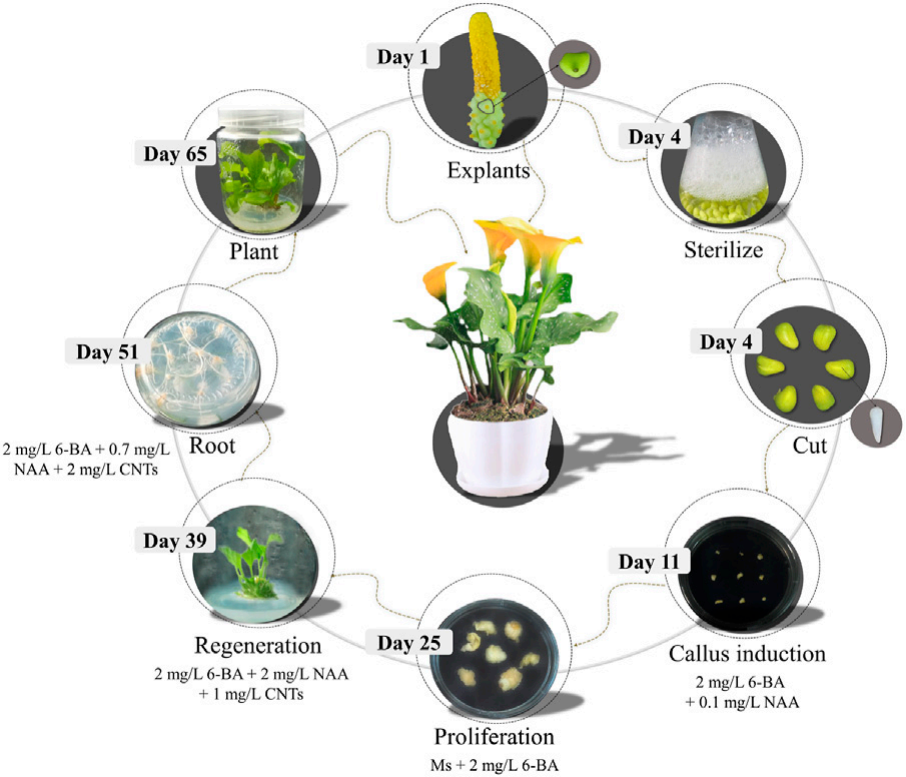
Schematic diagram of seed embryo regeneration system of calla lily.

Induction of embryonic callus and a robust *in vitro* regeneration system are the foundations of the stable genetic transformation of a calla lily. In this research, the optimal conditions for the induction and regeneration of embryogenic callus were identified for hybrid seeds between male “Black Magic” and female “Liming”. The seeds were harvested 60 days after pollination and kept at 4°C for 3 days. The embryonic tissues were then put on CIM medium and cultivated at 25°C for 3 weeks in the dark. It was allowed to form light yellow granular callus. In the EIM medium, an embryonic callus was identified and developed. When the callus reached a diameter of around 1–2 cm, it was transferred to the GM medium to stimulate germination, and subsequently to the RM medium to root. Step-by-step procedures could be demonstrated in Figure 5.

The species of explants is significant for embryogenic callus induction. In previous studies, only leaves and tubers were selected as explants for embryogenic callus induced in calla lily. Regeneration failed when leaf induction was used (Gong et al., 2008), and the induction efficiency was low when tubers were used (Duquenne et al., 2006). In most plants, leaf and stem explants are more difficult to sterilize than seed embryos (Deng et al., 2020), the same as in calla lily. The sterilization of calla lily tubers has also been an unsolved problem (Fan et al., 2005). Seed embryos play an essential role in *Triticum aestivum* (Tamimi and Othman, 2021), *P. ostii* (Liu et al., 2021) and other plants that are difficult to induce callus. So far, there are no reports of using seed embryos as explants in calla lily. In this study, the induction efficiency of embryogenic callus was increased by about 70% when seed embryos were selected as explants. Furthermore, the maturity of the seed embryo influences callus induction and regeneration. Here, callus induction and regeneration were carried out on calla lily seed embryos at different stages after pollination. The results showed that the induction rate increased gradually with the increase of maturity. However, most studies and reviews have shown that immature embryos are the best explant source for tissue culture (Yang et al., 2015; Deng et al., 2020; Shimizu-Sato et al., 2020). The reason for this difference may be related to the physiological state and differentiation degree of explants of different species (Abumhadi et al., 2005; Da Silva et al., 2015). In addition, ease of handling, a lack of seasonal limitations, and availability in bulk quantities are essential factors for selecting mature seed embryos for tissue culture (Yin et al., 2011; Ren et al., 2020).

Sane et al. (2012) found that callus induction and regeneration are genetically determined. The ability of different genotypes to induce and regenerate embryonic callus varies, resulting in genotypic dependence in genetic transformation (Yu et al., 2019). Consequently, each species may have a genotype-dependent optimal regeneration condition (Shimizu-Sato et al., 2020). In this study, the callus induction rate of hybrid seed embryos of “Carrera” and “Dynamo” was the lowest, and nearly no callus was obtained with the lowest induction rate. The callus induction rate of hybrid seed embryos of “Black Magic” and “Liming” reached 94%. Patterns of connection between genotype and induction rate have been developed (Shimizu-Sato et al., 2020). By transcriptome analysis (Zhang et al., 2019b), *WOX* genes, which have an original role in embryo development in seed plants and promote the regeneration of transformed callus, were particularly elevated in the high-regeneration lines as compared to the low-regeneration lines. Induced expression of *AtLEC2* triggers embryogenic callus development, but overexpression of *AtIPT7* or *AtIPT9* improves shoot regeneration without exogenous cytokinin (Li et al., 2019). Consequently, the discrepancies between genotypes may be due to the expression of distinct genes.

The establishment of transformation is highly dependent on the availability of calla lily somatic embryo induction, which is influenced by a number of PGRs. Auxin and cytokinin are vital hormones regulating the formation of plant callus. The proportion of auxin and cytokinin added to the culture medium affects plant organogenesis or somatic embryo formation (Tippani et al., 2019). The combination of auxin and cytokinin showed positive effects on inducing embryogenic callus, bud formation, and multi-bud formation in callus (Lee and Huang, 2014; Han and Kim, 2019), and is believed to influence the plant cell cycle and ability of morphogenesis (Jones et al., 2010). For example, auxin combined with 6-BA was effective in stimulating cv. ‘Fengdan’ somatic embryos (Ren et al., 2020). NAA and 6-BA are calla lily most prevalent PGRs combination (Fan et al., 2005). Consequently, 6-BA and NAA were chosen as the primary PGRs in this investigation, and their concentrations were tuned accordingly. The data suggest that the callus induction rate was maximal when 6-BA was at 2 mg/L and NAA was at 0.1 mg/L. It is important to note that the induction rate increased progressively with increasing 6-BA concentration. 6-BA is a crucial cytokinin for callus and shoot induction (Shen et al., 2015), including modulating shoot growth and proliferation, as well as stimulating cell multiplication and enlargement (Mok and Mok, 2001). It is hypothesized that the usage of cytokinin influences callus formation by reducing cell wall lignification, hence boosting *in vitro* callus initiation and development (Hoque et al., 2006). Nevertheless, the concentration should not be extremely high. When the concentration reached 2 mg/L, the induction ratio declined in this study. Research revealed that a high concentration of cytokinin inhibits callus cell division and that excessive cytokinin application may trigger herbicidal effects and impede the callus induction process (Song, 2014). As an auxin, the callus induction efficacy of NAA was reduced as its concentration increased in this study. When certain species are maintained *in vitro* with high auxin concentrations, they may undergo explant oxidation, a phenomenon frequently observed in palms (Moura et al., 2009). However, in *N. nucifera*, a low concentration of auxin was ineffective in inducing callus formation, while a moderate quantity of auxin mixed with 6-BA was most effective (Deng et al., 2020). This is comparable to the concentrations used for callus induction in rice (Weigel et al., 2002) and a classic tobacco model (Skoog and Miler, 1957). This is because the susceptibility of different plant species to PGRs varies. For example, 1.5 mg/L of synthetic auxin in *Allium sativum* and 2 mg/L in *Camellia sinensis* have been used to induce calli in plants (Chevala et al., 2016; Malik et al., 2020). Although the concentration of auxin was low during the callus induction stage, it was essential to enhance the concentration of NAA throughout the rooting and germination stages. The varied responses can be explained by PGR’s role in controlling the cell cycle as they regulate critical enzymes involved in the cell cycle, and exogenous application of PGR alters levels of other endogenous hormones, causing variation in the developmental process and variation in the dissipation of plant cell wall (Vergara et al., 2017). The balance between endogenous and exogenous PGRs plays an essential role *in vitro* culture (Kumari et al., 2018; Bidabadi and Jain, 2020; Kosakivska et al., 2020; Lardon and Geelen, 2020; Wang et al., 2021). On the other hand, PGRs regulate the formation and accumulation of ethylene and other phenolic compounds in plants during the closed phase of tissue culture, which in turn promotes the formation of necrotic cells and tissues on the explant material being cultured (Kumar et al., 2009). In this work, therefore, cultures at various developmental stages exhibited distinct responses to PGRs.

In this work, incorporating CNTs to devise the regeneration system had no discernible influence on the callus induction of calla lily, and the induction rate was greatest when no CNTs were present. In the *in vitro* cultivation of roses and date palms, however, the biomass and fresh weight of callus increased dramatically, as did the proliferation and differentiation of callus (Taha et al., 2016; Ghasempour et al., 2019; Scherwinski-Pereira et al., 2010). These findings demonstrated that CNTs promoted callus formation in several plant species, except calla lily. This may be related to the various susceptibilities of distinct species’ physiological conditions to CNTs. In addition to species differences, this may be owing to the greater absorption of minerals that are unsuitable for embryogenesis and reproduction. In this instance, the addition of CNTs enhanced germination and rooting. At the germination stage, the application of CNTs improved seedling differentiation. At the rooting stage, a high concentration of CNTs considerably enhanced the average root length (Wang et al., 2012). Reported that oxidized multi-walled carbon nanotubes greatly improved root cell elongation. It enhanced root and stem development, perhaps due to the absorption and accumulation of multi-walled CNTs by roots, followed by their transport to leaves (Smirnova et al., 2012). Similar findings were observed with radish, rapeseed, and lettuce (Lin and Xing, 2007).

In order to directly verify the transformation efficiency, we used *A. tumefaciens* containing pCAMBIA1304 vector to transfect the callus of *Zantedeschia*. *GUS,* as a reporter gene, could directly observe the transformation effect. *Agrobacterium* transfers its T-DNA into plant chromosomes during co-culture (Wu et al., 2015). Excessive growth of *A. tumefaciens* will cause browning and death of callus during co-culture. Previous studies showed that co-culture for 3 days significantly impacted the number of *GUS* positive callus in rice (Toki et al., 2006). In this study, co-culture for 3 days had the best effect. We choose 8 mg/L hygromycin, the same as the previous study in *Rosa chinensis* (Chen et al., 2016). In the early stage after *A. tumefaciens*, selected high Hygromycin concentration (50 mg/L) to suppress the excessive growth of *A. tumefaciens* on the callus surface, and the low hygromycin concentration (20 mg/L) did not affect the growth of rose plants in the later stage (Liu et al., 2021). The callus transformation process of *Zantedeschia* is usually carried out by adding cefotaxime to the culture medium (300 mg/L) to inhibit the overproduction of *A. tumefaciens*.

One of the main obstacles to extending the use of improved calla lily varieties in cultivation is their inefficient regeneration *via* callus. The procedure was optimized using an orthogonal design based on the *in vitro* regeneration-influencing factors genotype, PGRs, and CNTs. Here, a very effective regeneration system employing mature calla lily embryos was established. With the indirect somatic embryogenesis system, mature embryos may be employed as target materials for the transformation and seedling production of calla lily. On this premise, the *GUS* reporter gene was used to examine the genetic transformation system in an exploratory manner. It establishes the groundwork for modern calla lily breeding.

## Data Availability

The original contributions presented in the study are included in the article/Supplementary Material; further inquiries can be directed to the corresponding authors.
